# *Withania somnifera* Root Extract Has Potent Cytotoxic Effect against Human Malignant Melanoma Cells

**DOI:** 10.1371/journal.pone.0137498

**Published:** 2015-09-03

**Authors:** Babli Halder, Shruti Singh, Suman S. Thakur

**Affiliations:** CSIR- Centre for Cellular and Molecular Biology (CSIR-CCMB), Uppal Road, Telengana-500007, India; Duke University Medical Center, UNITED STATES

## Abstract

In Ayurveda, *Withania somnifera* is commonly known as Ashwagandha, its roots are specifically used in medicinal and clinical applications. It possesses numerous therapeutic actions which include anti-inflammatory, sedative, hypnotic and narcotic. Extracts from this plant have been reported for its anticancer properties. In this study we evaluated for the first time, the cytotoxic effect of *Withania* root extract on human malignant melanoma A375 cells. The crude extract of *Withania* was tested for cytotoxicity against A375 cells by MTT assay. Cell morphology of treated A375 cells was visualized through phase contrast as well as fluorescence microscopy. Agarose gel electrophoresis was used to check DNA fragmentation of the crude extract treated cells. Crude extract of *Withania* root has the potency to reduce viable cell count in dose as well as time dependent manner. Morphological change of the A375 cells was also observed in treated groups in comparison to untreated or vehicle treated control. Apoptotic body and nuclear blebbing were observed in DAPI stained treated cells under fluorescence microscope. A ladder of fragmented DNA was noticed in treated cells. Thus it might be said that the crude water extract of *Withania somnifera* has potent cytotoxic effect on human malignant melanoma A375 cells.

## Introduction

*Withania somnifera* (Ashwagandha) is a plant used in the traditional Ayurvedic and Unani medical system also. It is a common ingredient for treating a variety of musculoskeletal conditions and is preferred as a tonic to augment energy, develop overall health and during pregnancy [1–2]_._ It can generate a state of non-specific increased resistance (SNIR) to the adverse effects acquired from different physical, chemical, and biological agents [3]. They do not have a recognized specific mechanism of action but can counteract various pathological conditions [4].

Cancer is a hyperproliferative disorder that leads to uninhibited proliferation, dysregulation of apoptosis and cell cycle. *Withania somnifera* components are found to be effective against several types of cancers. It has been observed that *Withania somnifera* extract can inhibit activities of key TCA cycle enzymes like isocitrate dehydrogenase, malate dehydrogenase in colon cancer bearing animals [5]. 1-oxo-5beta, 6beta-epoxy-witha-2-enolide isolated from the roots of *Withania somnifera*, is effective against ultraviolet radiation induced skin carcinoma in rats [6]. Singh et al reported that, *Withania somnifera Dunal* has anti-neoplastic activity against urethane induced lung-adenomas in adult male albino mice [7]. Withaferin A, a major chemical constituent of *Withania somnifera*, induces mitochondrial dysfunction in human leukemia HL-60 cells and activates both mitochondrial dependent and independent apoptotic pathways [8]. *Withania* extracts have shown anti-proliferative activity against MCF -7, pancreatic, prostate, renal and fibrosarcoma cells [9–13].

Thus it can be said that, constituents isolated from *Withania somnifera* play a significant role against several kind of neoplastic growth and hence might be used as an alternate chemotherapeutic agent.

## Results

### Crude water extract of *Withania somnifera* showed reduction in viability of A375 cells

**Fig 1:**
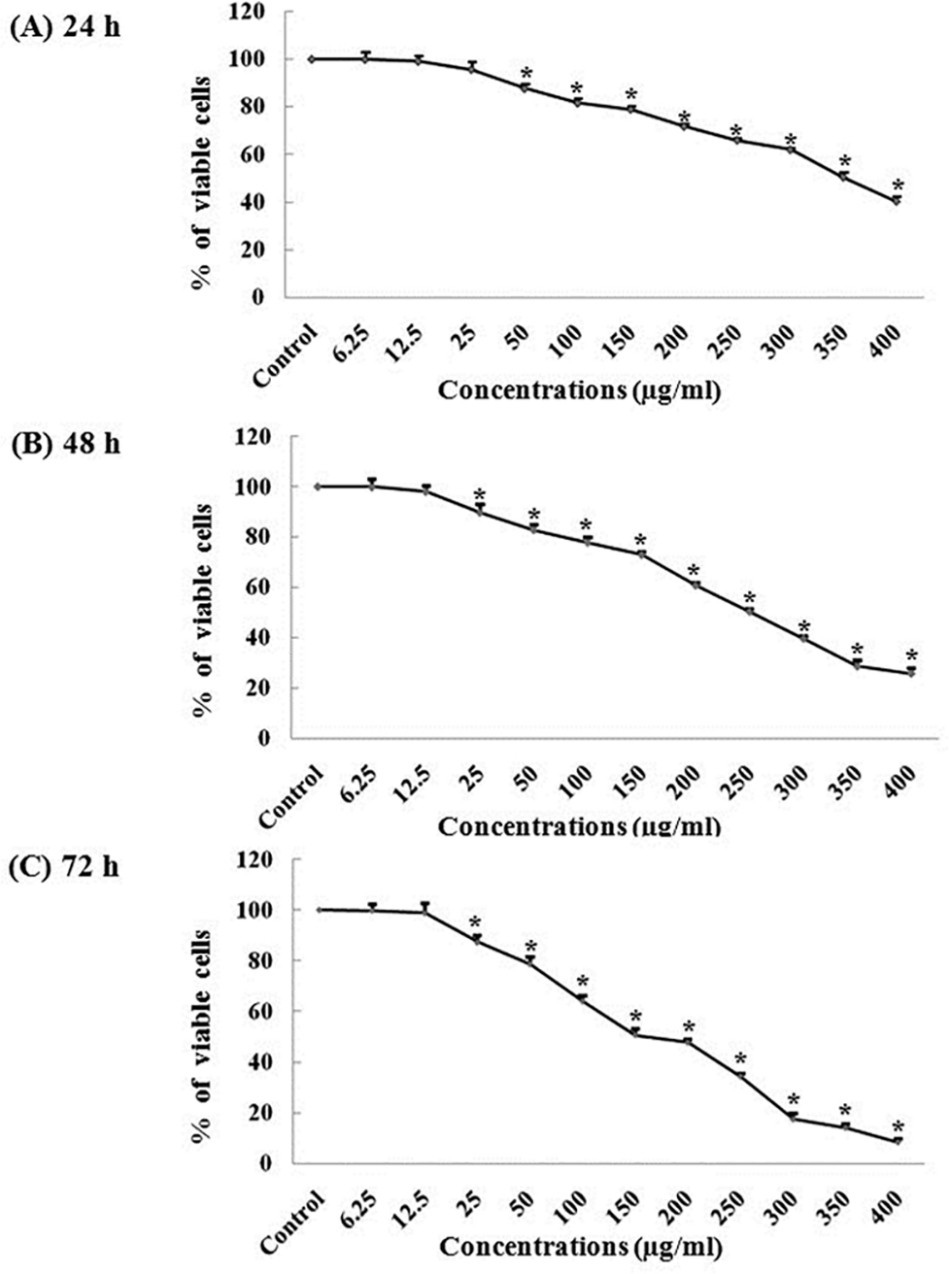
Different concentrations of crude water extract of *Withania* showed cytotoxic effect on A375 cell line at different time point of incubation. Cells were treated with different concentrations (6.25,12.5,25,50,100,150,200,250,300,350,400μg/ml) of *Withania* for 24 hr (A), 48 hr (B) and 72 hr (C). All data are expressed as mean ± SD of three independent experimental observations.

MTT assay was performed to evaluate the cytotoxic effect of *Withania* crude extract on A375 cells. Cells were treated with different concentrations of *Withania* (6.25, 12.5, 25, 50, 100, 150, 200, 250, 300, 350 and 400μg/ml) for 24, 48 and 72 hr. A significant reduction of cell viability was seen in dose and time dependent manner when compared with the control or vehicle treated A375 cells. The calculated IC_50_ value for 24 hr is 350μg/ml in A375 cells (Fig 1A), for 48hr is 250μg/ml (Fig 1B) and 200μg/ml for 72 hr of incubation (Fig 1C).

### Phase contrast microscopic observations of A375 cells after treatment with *Withaniasomnifera* crude water extract

**Fig 2:**
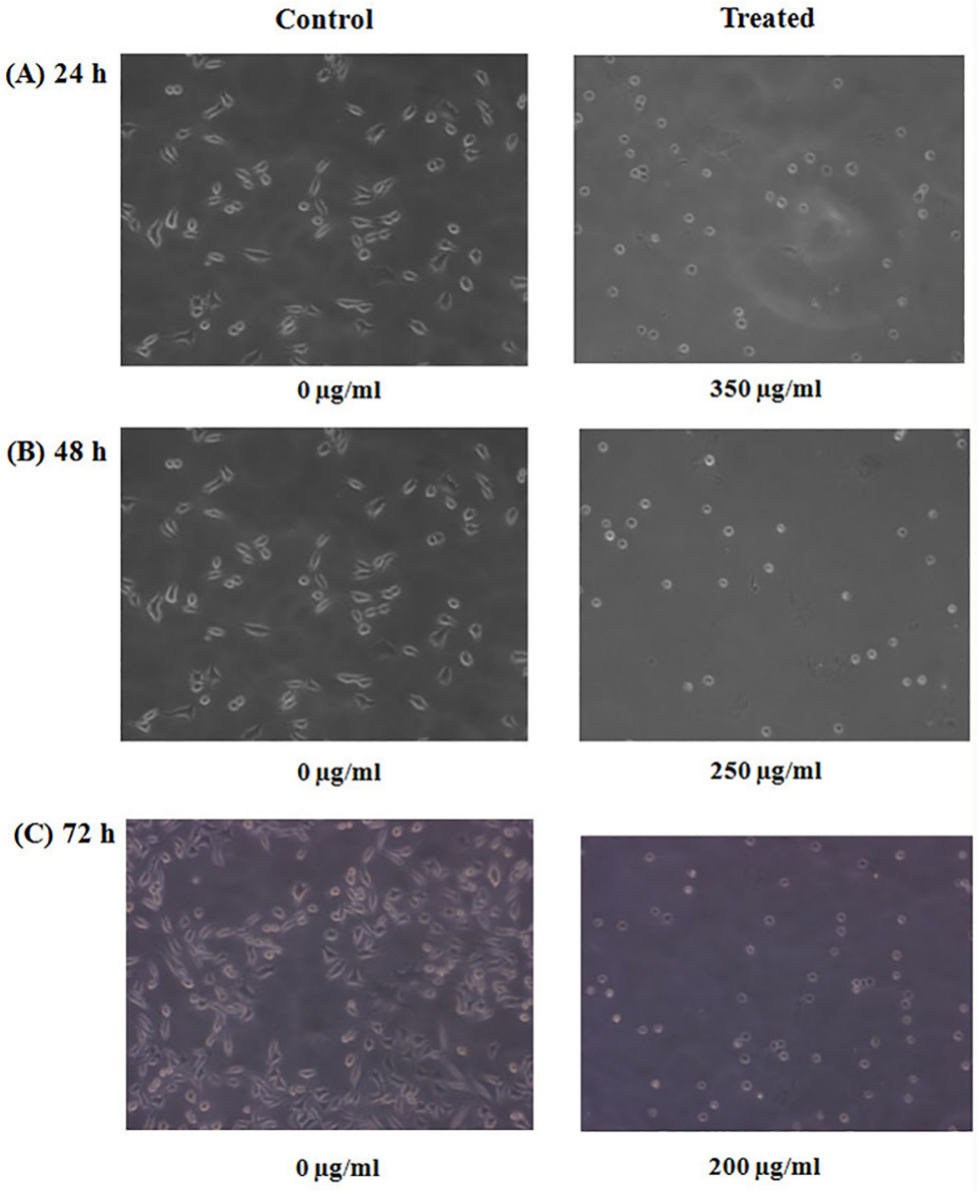
Morphological changes observed in A375 treated cells. Cells were treated with 350, 250 and 200 μg/ml of *Withania* water crude extract for 24, 48 and 72 hr of incubation respectively.

A375 cells were treated with IC_50_ concentrations (350, 250 and 200μg/ml) from each time point (24, 48 and 72hr) and observed under a phase contrast microscope. Morphological alterations were observed in treated A375 cells in comparison to the control or vehicle treated. In case of control cells the shape of A375 cell is polygonal but after treatment with different concentrations the shape became spherical (Fig 2).

### Fluorescence microscopic observations of A375 cells after treatment with *Withania somnifera* crude water extract

**Fig 3:**
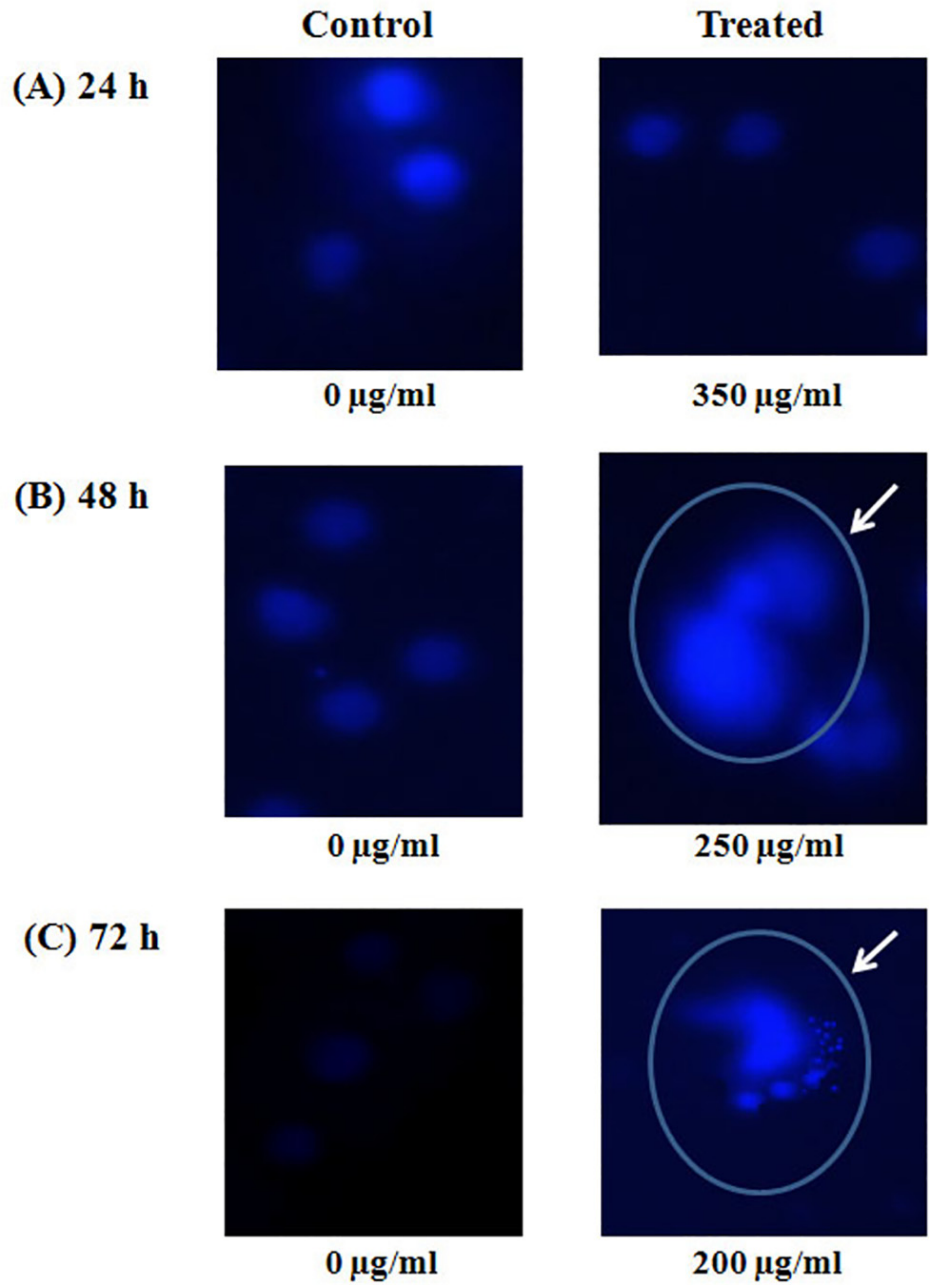
Morphological changes observed in DAPI stained A375 treated cells. Cells were treated with 350, 250 and 200 μg/ml of *Withania* water crude extract for 24, 48 and 72 hr of incubation respectively. (A) 24 hr treatment, B) 48 hr treatment with circle and arrow denoting the nuclear blebbing and (C) 72 hr treatment with circle and arrow denoting the nuclear fragmentation.

A375 cells were treated with respective IC_50_ concentrations of *Withania* fo*r* 24, 48 and 72 hr. The cells were then treated with DAPI to see the nucleus of control and treated cells. After 24 hr of incubation there was no change in nucleus (Fig 3A). But in case of 48 hr of incubation, nuclear blebbing was observed as shown in the figure by circle and arrow (Fig 3B). Further apoptotic bodies (denoted by circle and arrow) were seen in case of 72 hr of incubation (Fig 3C).

### *Withania* crude extract treatment induced DNA fragmentation

**Fig 4:**
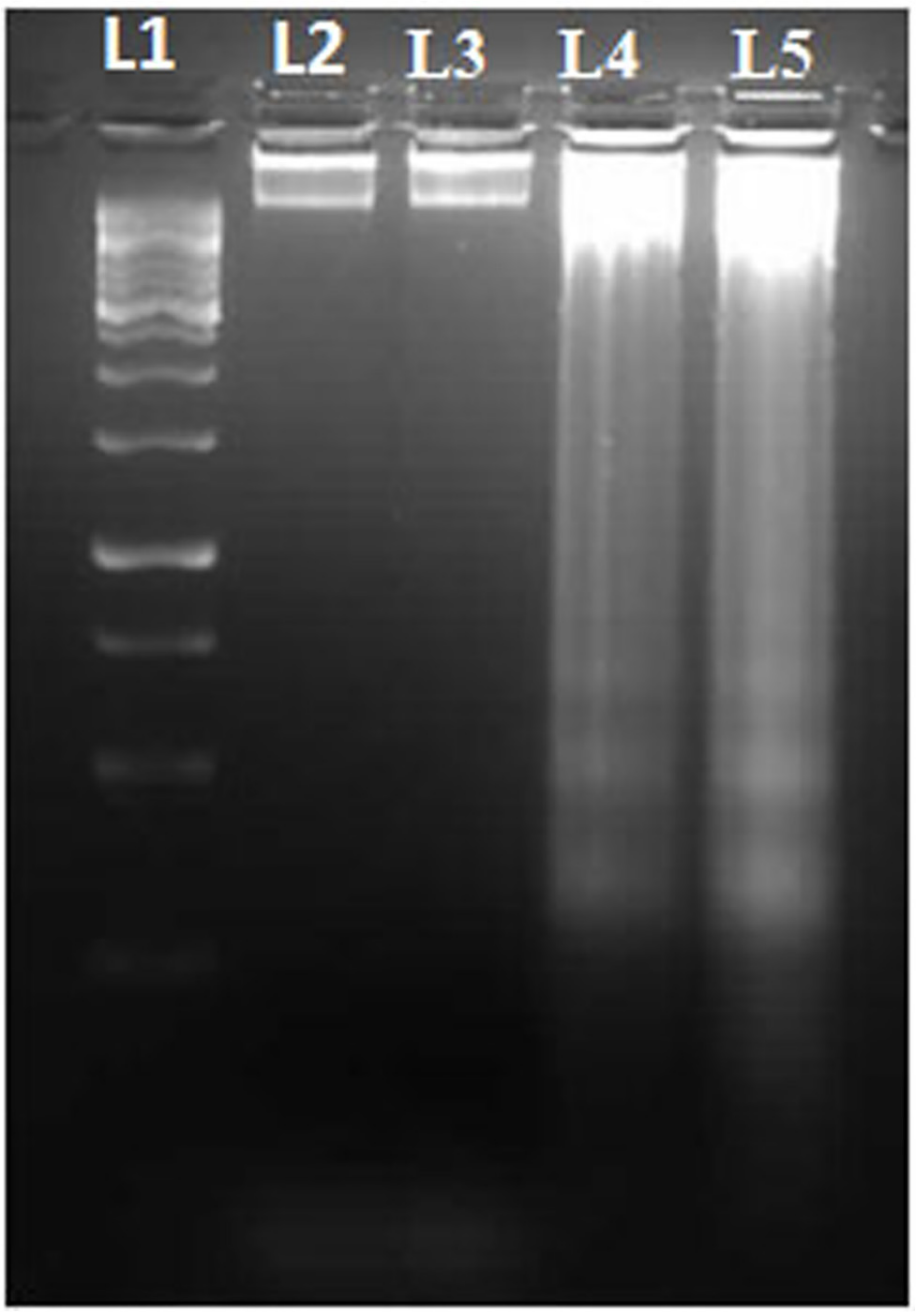
*Withania* crude water extract treatment induces DNA fragmentations. L denotes lane in the gel, L1 = 1kb DNA ladder, L2 = control (0 μg/ml), L3 = 24 hr treatment with 350 μg/ml, L4 = 48 hr treatment with 250 μg/ml and L5 = 72 hr treatment with 200 μg/ml.

DNA fragmentation is one of the hallmark sign of apoptosis. DNA fragmentation assay was carried out in A375 cells after 24, 48 and 72 hr of incubation. Our experimental results demonstrated a distinct ladder formation in 48 and 72 hr of *Withania* crude extract treated cells as compared to untreated or vehicle treated cells (Fig 4). However no DNA fragmentation could be seen in the treated cells upon 24 hr incubation with *Withania* crude extract.

### Electro spray ionization mass spectrometry (ESI-MS) analysis of water extract of *Withania* root

**Fig 5:**
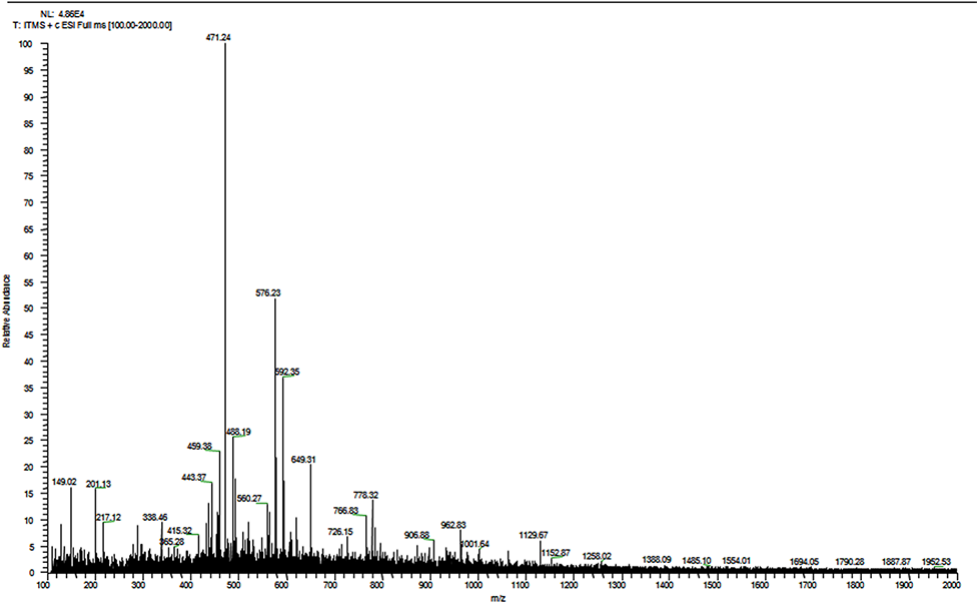
ESI-MS spectra of water extract of root from *Withania somnifera*.

To characterize the compounds in water extract of *Withania* root we have performed ESI- MS experiments (Fig 5). ESI-MS spectra have been recorded from m/z 100 to 2000. The major peak observed at m/z 471.2, 576.2, 592.3, 488.1, 459.3, 649.3, 443.3, 778.3, 149.0, 962.8, 201.1 and 1129.6 according to the relative abundance. It was observed that major component in the water extract was Withaferin-A (Molecular weight 470, Molecular formula-C_28_H_38_O_6,_ [M+H]^+^ = 471).

## Discussion

Cancer is characterized by uncontrolled cell growth which involves transformation, dysregulation of apoptosis, proliferation, invasion, angiogenesis and metastasis [14]. Among the different skin cancers melanoma is the most aggressive one and it is the reason for about 75% of death due to skin cancer [15]. There are several drugs available in market to treat melanoma however none are target specific and show lots of side effects. As a result, scientists are exploring the traditional and presumably safe natural medicines for the anticancer therapeutics.

In Ayurveda, *Withania somnifera* (Ashwagandha) is a traditional plant and it is extensively used as a medication throughout India. Ingredients of this medicinal plant have been formulated in many ways and used against arthritis and rheumatism to improve overall health conditions [16]. *Withania somnifera* is well identified for its various biological activities such as adaptogenic/anti-stress, immunomodulatory, anti-ageing, anti-fatigue, antioxidant, anti-parkinsonism, antiulcerogenic and anti-tumors/adenomas properties [4, 16–26]. A number of studies have been carried out to assess the efficiency of *Withania somnifera* in prevention and treatment of diverse kinds of cancer of colon [5], lung [7], blood [8], breast [11], renal [12], prostate [11], pancreatic [10] and mouse skin [6]. Most of the skin cancer studies have been carried out on the mouse model and there is no report about the effect of *Withania somnifera* on human malignant melanoma cells, A375.

In this present study, the cytotoxic effect of *Withania somnifera* crude water extract has been evaluated on human malignant melanoma cells, A375. In MTT assay we found a significant reduction of cell viability in case of treated cells (for 24, 48 and 72 hr) as compared to that of the control or vehicle treated cells. The calculated IC_50_ values for 24hr are 350μg/ml, for 48 hr is 250 μg/ml and for 72 hr is 200μg/ml. So from this data it can be said that reduction of the cell viability is a dose as well as time dependent phenomena due to *Withania somnifera* crude extract treatment. This result can be co-related with the morphological changes after treatment with *Withania*. Both phase contrast and fluorescence microscopic observations demonstrated the morphological differences between *Withania somnifera* crude water extract treated and untreated cells. DAPI stained treated cells showed nuclear blebbing and apoptotic bodies which are the evidences of apoptotic induction. But in case of un-treated cells DAPI staining showed intact nucleus. So we had further extended our study to check whether different IC_50_ concentrations at different time point treatment could induce DNA fragmentation or not. DNA fragmentation is a hallmark sign of late apoptosis brought about by inter-nucleosomal cleaving during the programmed cell death. We have found a clear ladder formation on agarose gel in case of treated samples. However there is no evidence of DNA ladder formation in case of control and 24 hr treated cells. So it can be said the DNA fragmentation initiated at 48 hr of incubation could be due to a late-apoptotic change. Both the microscopic and DNA fragmentation assays were indicating that *Withania somnifera* crude water extract might initiate apoptosis in A375 cells. So it could be said that induction of apoptosis might be one of the explanations for *Withania somnifera* crude water extract mediated death of A375 cells. Mass spectrometry experiment revealed the presence of more than 10 compounds in the water extract of *Withania somnifera*. Notably major component of this extract is Withaferin A.

To conclude, it might be said that the *Withania somnifera* crude water extract has potent cytotoxic effect on human malignant melanoma A375 cells. Both the microscopic observation and DNA fragmentation assays supported the initiation of apoptosis in treated cells. Our experiments showed an increment of apoptotic cell death in treated cells thereby suggesting a potent chemotherapeutic effect of *Withania somnifera* crude water extract. However further studies are required to identify the molecular mechanism of action of *Withania somnifera* in A375 cells. To the best of our knowledge, this is the first report of induction of apoptosis by *Withania somnifera* crude water extract treatment in human malignant melanoma A375 cells.

## Material and Methods

### Extraction method

*Withania somnifera* root powder (Ashwagandha Churna) was purchased from Dabur India Ltd, Alwar, Rajasthan (Pin-301030), India, (Lot No. 1223091097/ALW/00). Five gram of dried powder of root of *Withania somnifera* was taken and soaked overnight in 50ml of deionized water. After that it was stirred with the help of magnetic stirrer continuously for about 72 hr followed by centrifugation. The supernatant was then used for further experimentation.

### Cell culture

Human malignant melanoma cell line, A375 cell line was obtained from the National Centre for Cell Sciences (NCCS, Pune). The cell line was cultured in DMEM, supplemented with 10% fetal bovine serum, 25U/ml penicillin and 25 mg/ml streptomycin (Invitrogen Corporation, Grand Island, NY) at 37°C in a water-saturated atmosphere of 95% air and 5% CO_2_.

### MTT assay

The effect of crude water extract of *Withania somnifera* on A375 human malignant melanoma cell line was observed by MTT (3-(4,5-dimethylthiazol-2-yl)-2,5-diphenyl tetrazolium bromide) assay. MTT reagent was purchased from Calbiochem (La Jolla, CA). Cells were counted on haemocytometer and 5000 cells/well were plated in 96 well plate in 100μl of complete media (containing 10% of FBS).

Cells were treated with different concentrations (6.25, 12.5, 25, 50, 100, 150, 200, 250, 300, 350 and 400μg) of *Withania somnifera* for 24, 48 and 72 hr. After the stipulated time, MTT solution (5mg/ml) was added to each well and incubated for 3hr. The purple-colored precipitate of formazan was dissolved in 150 μl of DMSO (Sigma-Aldrich) by proper mixing. The color absorbance of each well was recorded at 570 nm in a Bio-Rad microplate reader with a reference serving as blank. Then, IC_50_ value of *Withania somnifera* crude extract was calculated.

### Morphological study under phase contrast and fluorescence microscope

#### Phase contrast microscopy

A375 cells were treated with different IC_50_ concentrations (350, 250 and 200 μg/ml) of water crude extract of *Withania somnifera* for 24, 48 and 72 hr. Following incubation, the cells were observed under phase contrast microscope at 20x magnification. Pictures were acquired with the help of Axiovision software.

#### DAPI (4’, 6-Diamidino-2-phenylindole) staining

A375 cells were treated with IC_50_ concentration (350, 250 and 200 μg/ml) of water crude extract of *Withania somnifera* for 24, 48 and 72 hr. After the incubation, cells were washed with PBS and then fixed with formaldehyde solution. After fixation, A375 cells were incubated with DAPI and observed under the fluorescence microscope.

### DNA fragmentation assay

A375 cells were treated for 24, 48 and 72 hr with their respective IC_50_ concentrations (350, 250 and 200 μg/ml) of water crude extract of *Withania somnifera*. After incubation, cells were washed and lysed in lysis buffer containing Tris, EDTA, SDS, proteinase K. DNA was extracted by following the procedure of phenol/chloroform/isoamyl alcohol extraction and was precipitated by ethanol. The precipitated DNA was dissolved in Tris-EDTA and then treated with RNAse A. After that DNA was run on 1.5% agarose gel stained with ethidium bromide for analysis.

### Statistical analysis

MTT Data were analyzed by using Graphpad Instat Software. All data are expressed as mean ± SD of three independent experimental observations. One-way ANOVA was applied to determine the difference between untreated control and *Withania* treated group. Dunnett’s multiple comparison test [27] was used as a posttest for determination of the significant levels *p<0.01.

### Electro spray ionization mass spectrometry (ESI-MS)

ESI-MS experiments (direct injection) were carried out on an ESI-Mass spectrometer with a linear ion trap mass analyzer (LTQ-IT, Thermo Finnigan, Waltham, MA). Spectra were obtained in positive ion mode by direct injection of water extract of *Withania* samples into the system using a syringe pump operated at a flow rate of 200 μLh^-1^. The electrospray voltage was set at 4.0 kV, and the capillary temperature was set at 200°C.
