# Correction: *Withania somnifera* Root Extract Has Potent Cytotoxic Effect against Human Malignant Melanoma Cells

**DOI:** 10.1371/journal.pone.0141053

**Published:** 2015-10-30

**Authors:** Babli Halder, Shruti Singh, Suman S. Thakur

In Fig 2B, the incorrect image was used for the 0 μg/ml Control at 48h. The image for the Control sample in Fig 2B incorrectly appears as a duplicate of the Control sample for Fig 2A. The authors have provided a corrected version of Fig 2 here. The authors have also provided uncropped images as Supporting Information.

**Fig 2:**
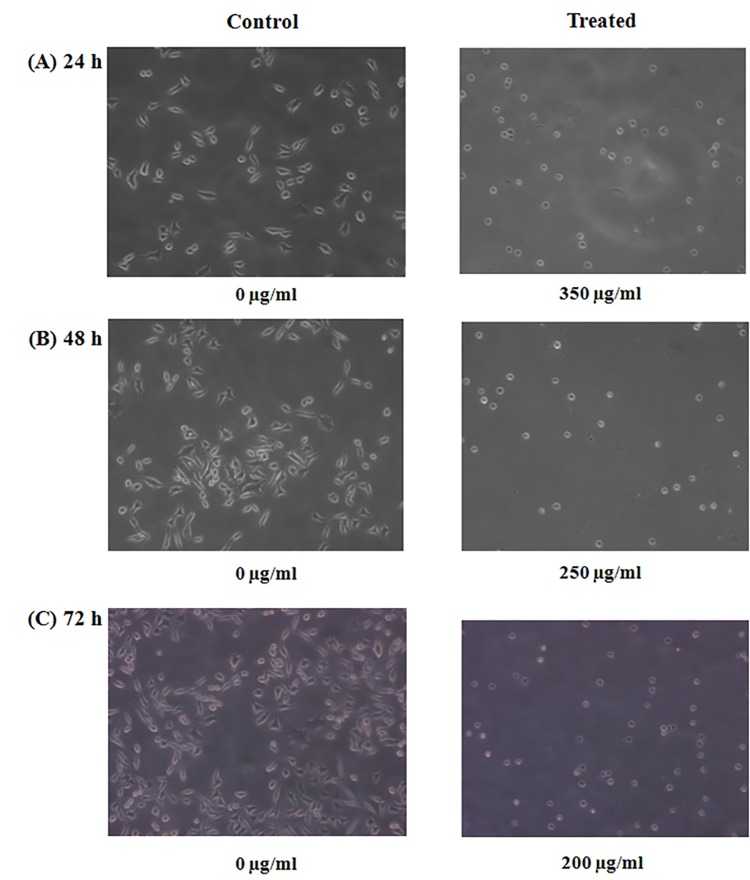
Morphological changes observed in A375 treated cells. Cells were treated with 350, 250 and 200 μg/ml of *Withania* water crude extract for 24, 48 and 72 hr of incubation respectively.

## Supporting Information

S1 File Uncropped images for Fig 2. (PPT)
