# Supplementary material for: Correction: Withania somnifera Root Extract Has Potent Cytotoxic Effect against Human Malignant Melanoma Cells
Source: PLoS One. 2015 Oct 30;10(10):e0141053. doi: 10.1371/journal.pone.0141053 (PMC4627760; doi:10.1371/journal.pone.0141053)
Supplement: S1 File — (PPT) [file pone.0141053.s001.ppt]

## Slide 1
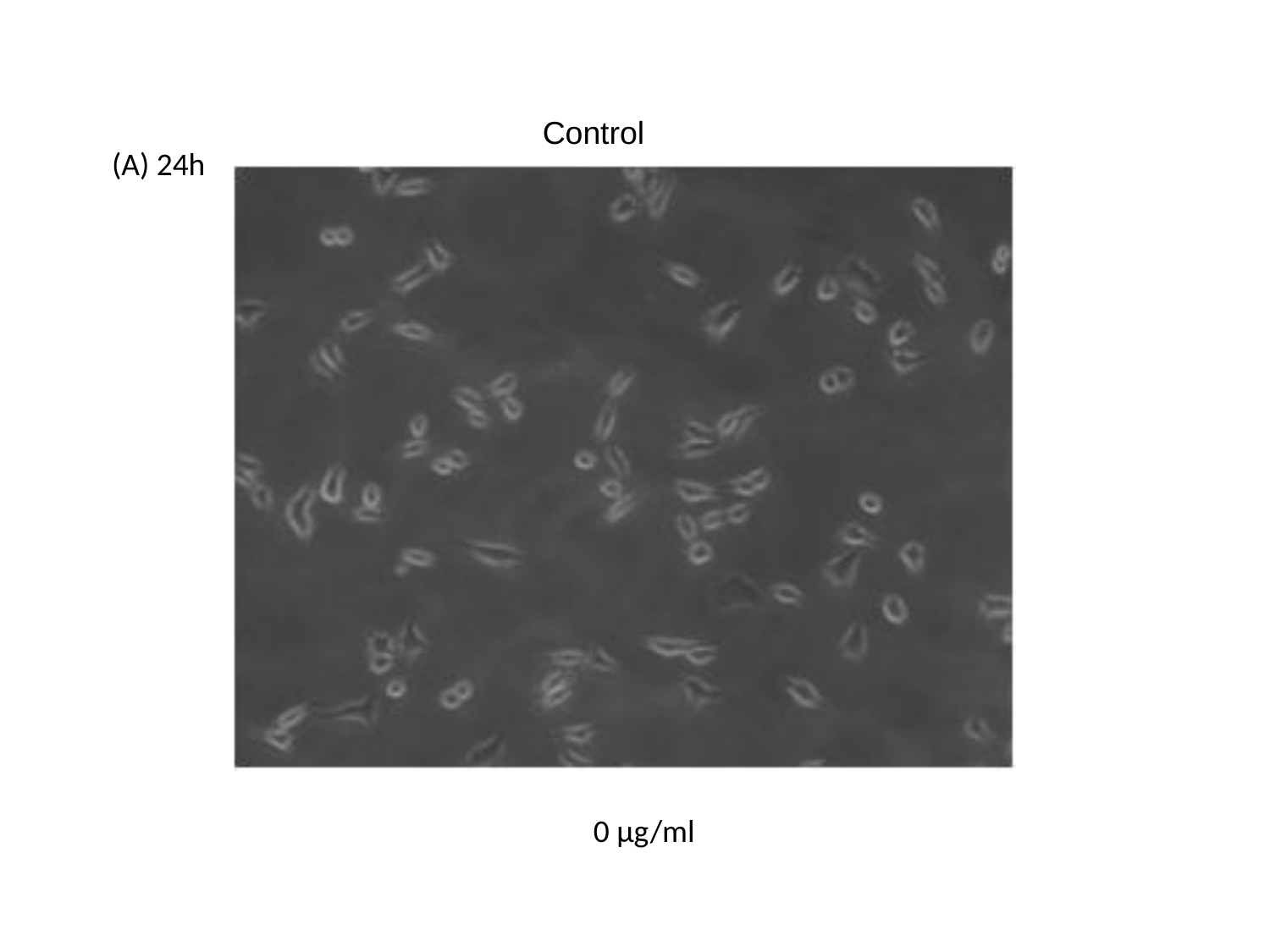

Control
 (A) 24h
0 µg/ml

## Slide 2
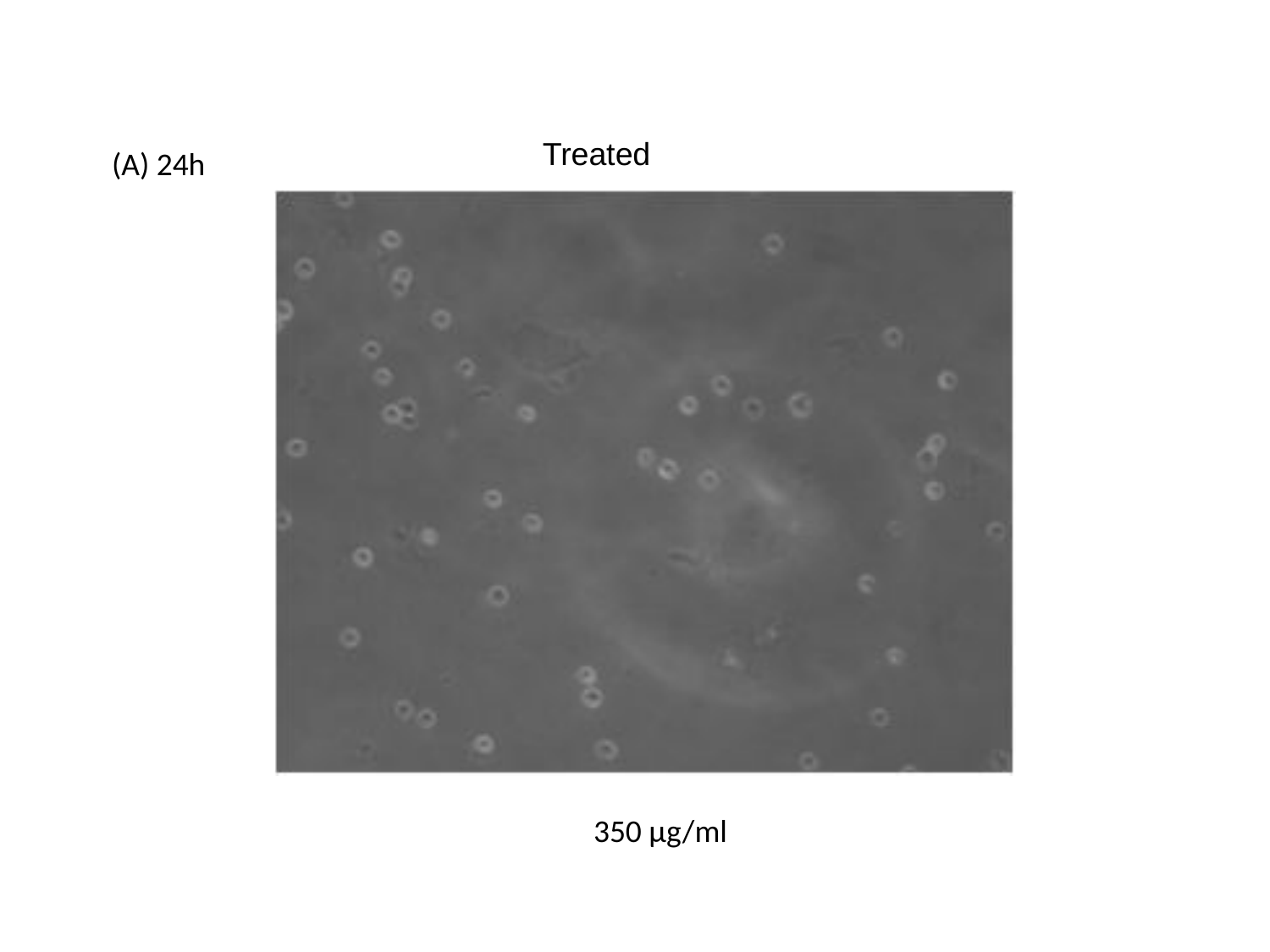

Treated
 (A) 24h
350 µg/ml

## Slide 3
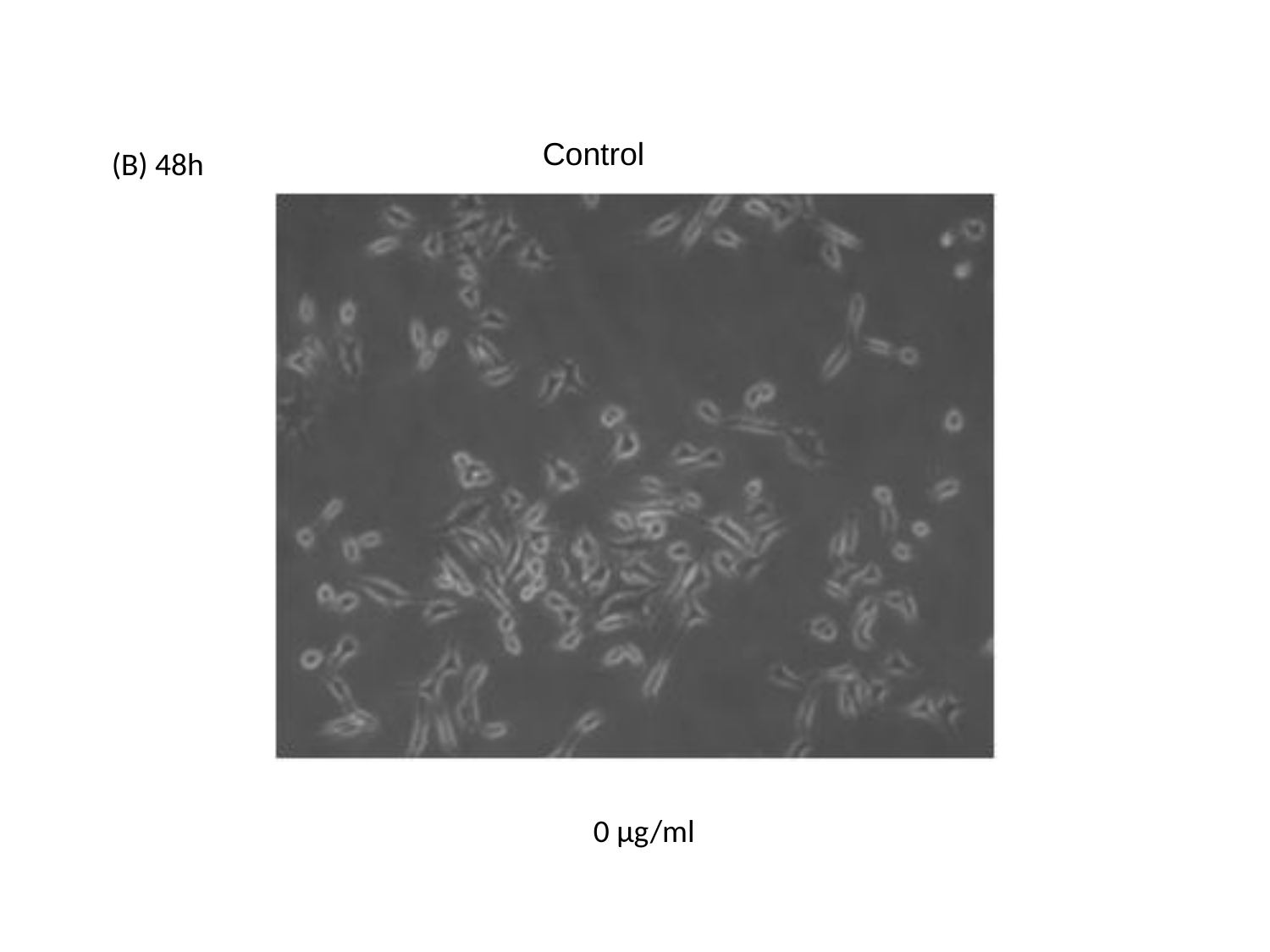

Control
 (B) 48h
0 µg/ml

## Slide 4
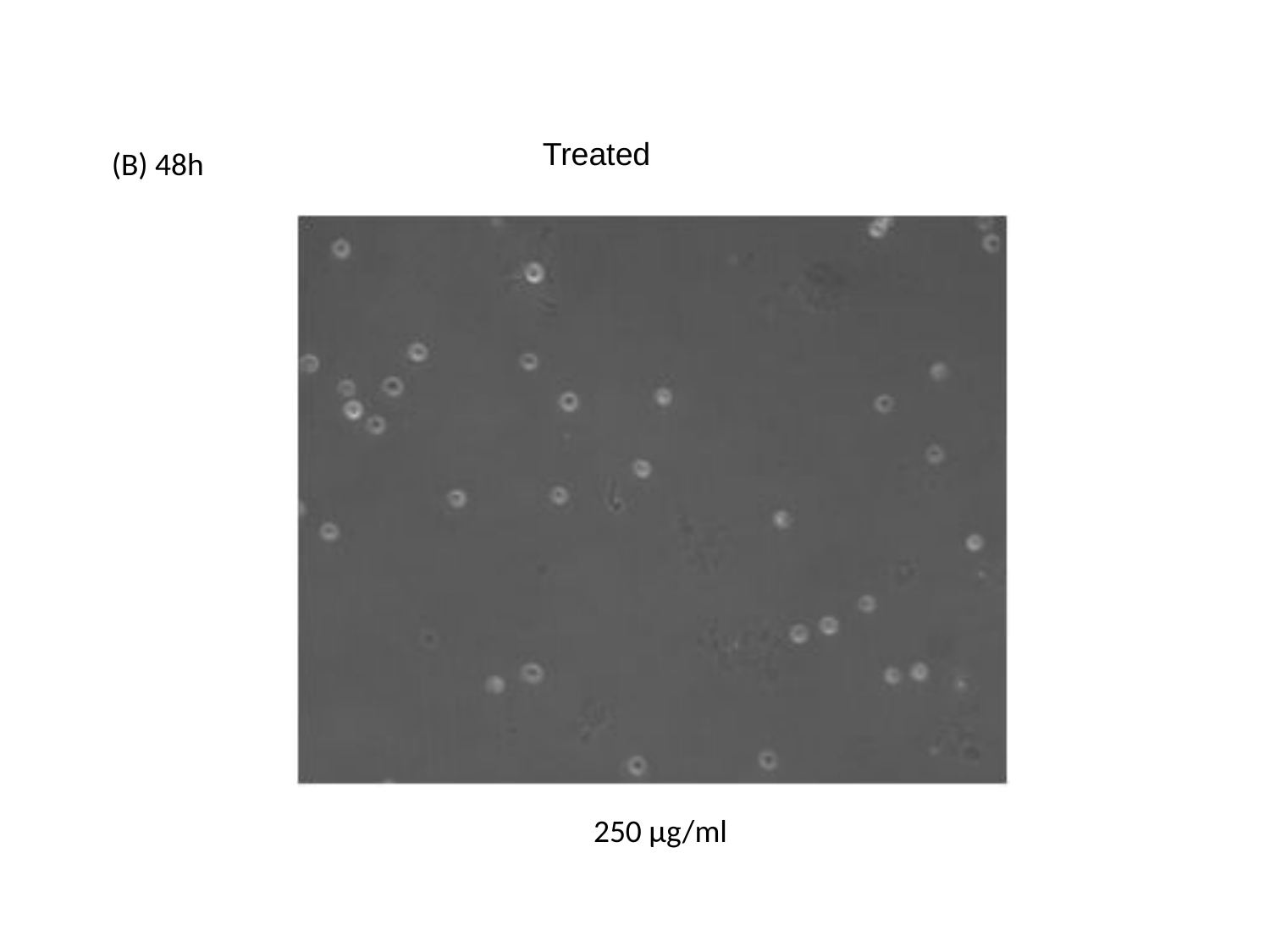

Treated
 (B) 48h
250 µg/ml

## Slide 5
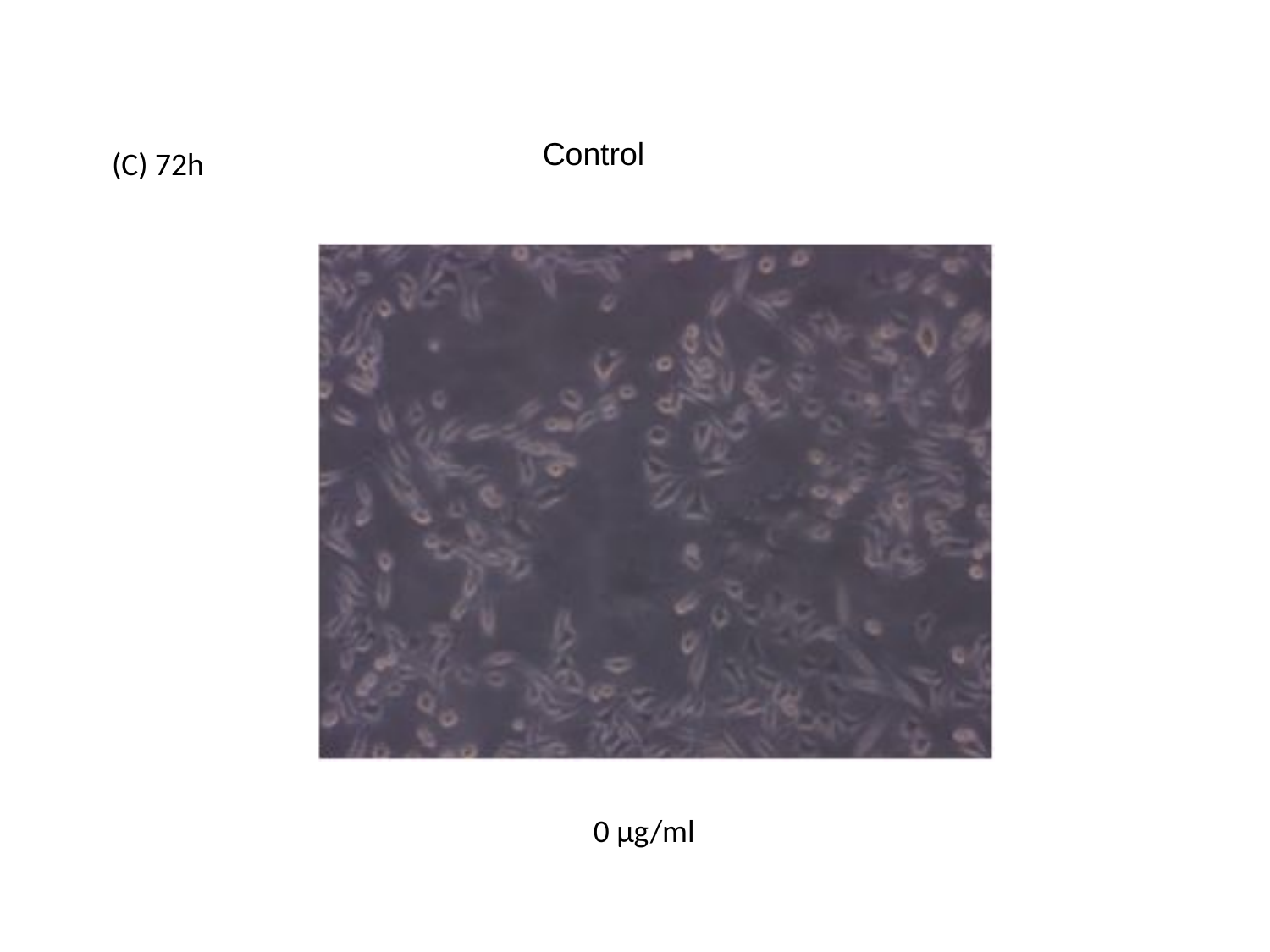

Control
 (C) 72h
0 µg/ml

## Slide 6
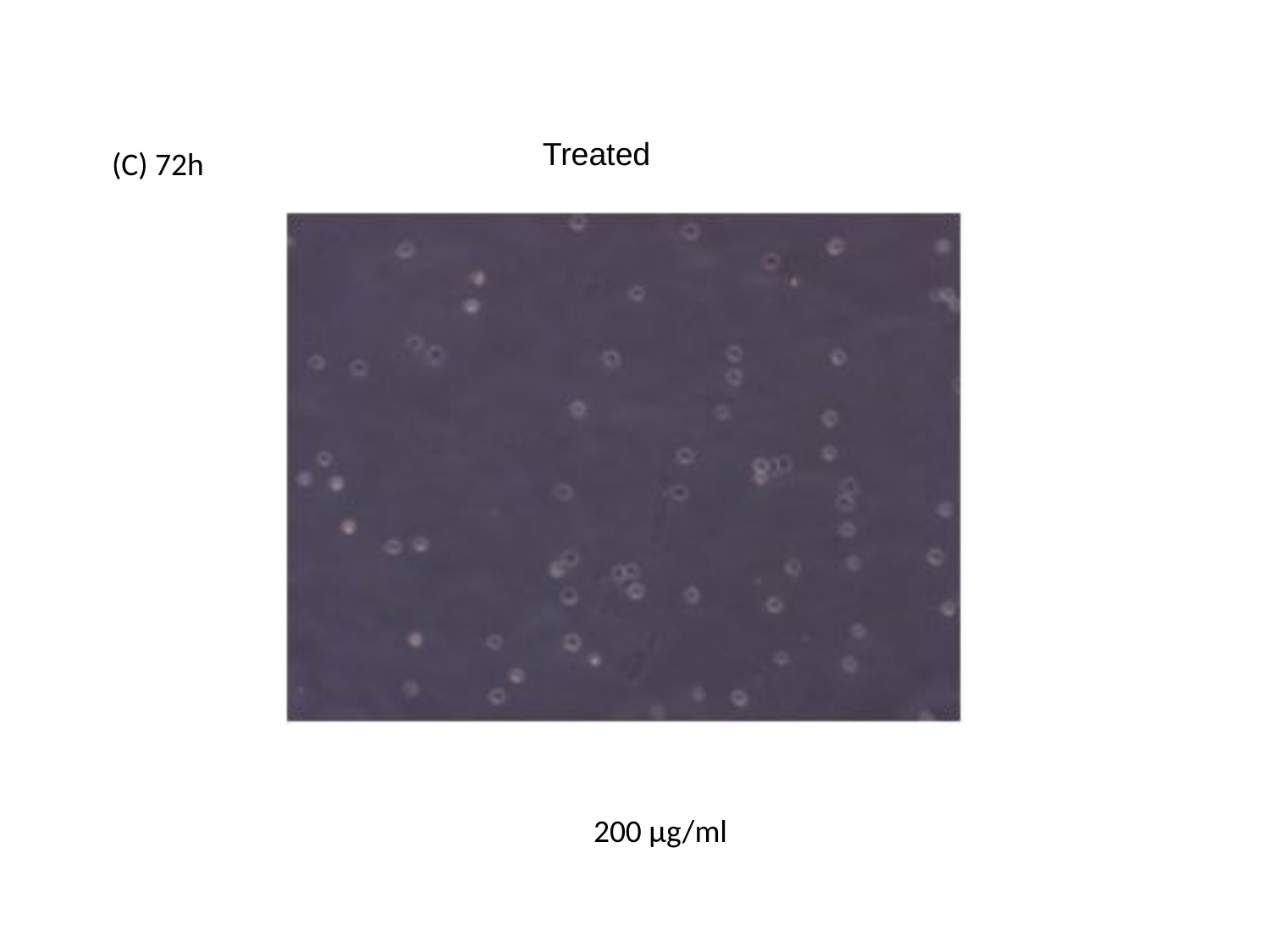

Treated
 (C) 72h
200 µg/ml

## Slide 7
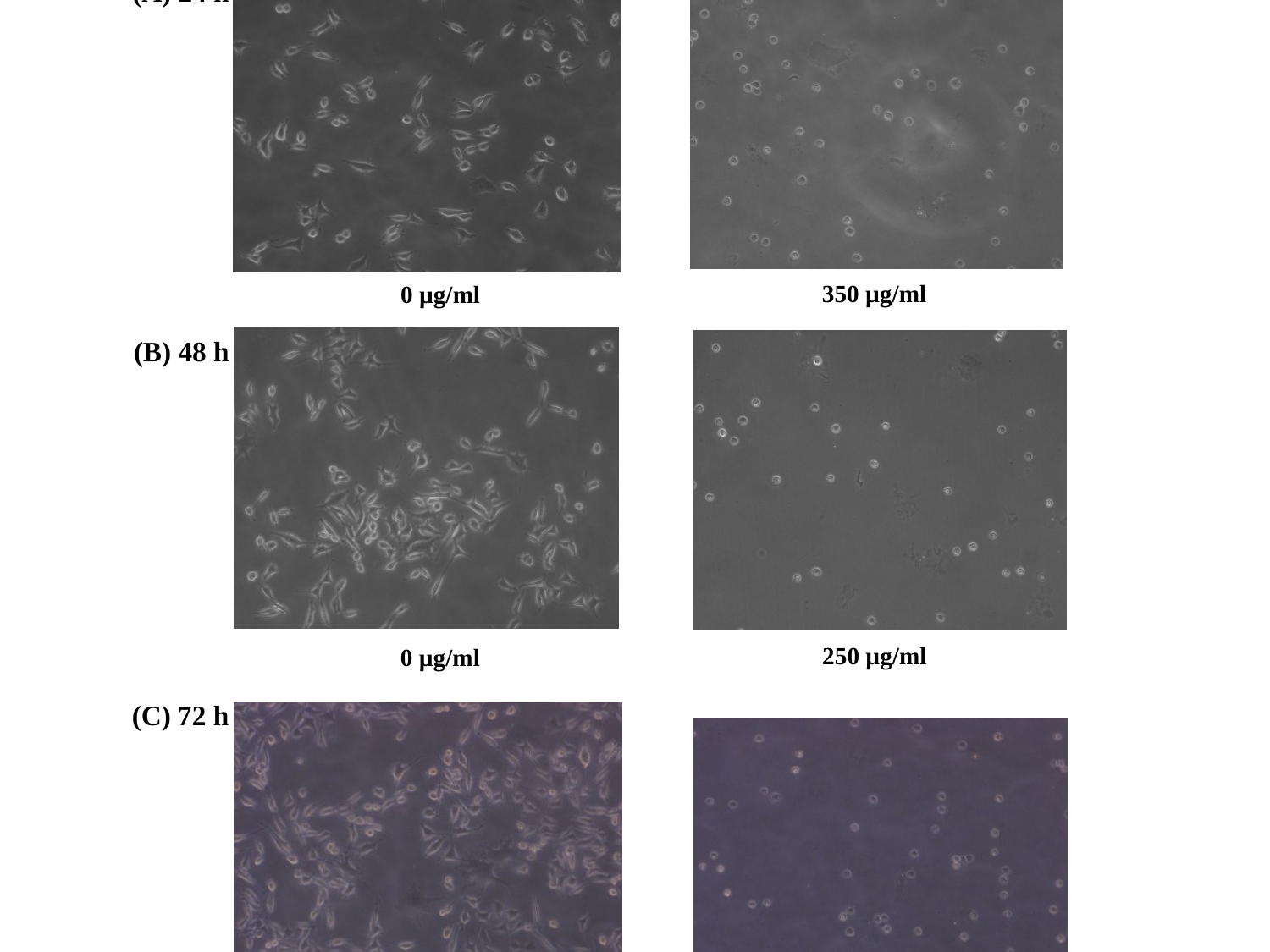

Treated
Control
 (A) 24 h
 350 µg/ml
 0 µg/ml
 (B) 48 h
 250 µg/ml
 0 µg/ml
 (C) 72 h
 200 µg/ml
 0 µg/ml
